# Genome-Wide Association Identifies Risk Pathways for SAPHO Syndrome

**DOI:** 10.3389/fcell.2021.643644

**Published:** 2021-03-18

**Authors:** Ruikun Cai, Yichao Dong, Mingxia Fang, Yuxuan Fan, Zian Cheng, Yue Zhou, Jianen Gao, Feifei Han, Changlong Guo, Xu Ma

**Affiliations:** ^1^National Research Institute for Family Planning, Beijing, China; ^2^National Human Genetic Resources Center, Beijing, China; ^3^Graduate School of Peking Union Medical College, Beijing, China; ^4^Beijing Chao-Yang Hospital, Capital Medical University, Beijing, China

**Keywords:** SAPHO syndrome, GWAS, WES, pathway analysis, immune-mediated conditions

## Abstract

SAPHO syndrome is a rare chronic inflammatory disease which is characterized by the comprehensive manifestations of bone, joint, and skin. However, little is known about the pathogenesis of SAPHO syndrome. A genome-wide association study (GWAS) of 49 patients and 121 control subjects have primarily focused on identification of common genetic variants associated with SAPHO, the data were analyzed by classical multiple logistic regression. Later, GWAS findings were further validated using whole exome sequencing (WES) in 16 patients and 15 controls to identify potentially functional pathways involved in SAPHO pathogenesis. In general, 40588 SNPs in genomic regions were associated with *P* < 0.05 after filter process, only 9 SNPs meet the expected cut-off *P*-value, however, none of them had association with SAPHO syndrome based on published literatures. And then, 15 pathways were found involved in SAPHO pathogenesis, of them, 6 pathways including osteoclast differentiation, bacterial invasion of epithelial cells, *et al.*, had strong association with skin, osteoarticular manifestations of SAPHO or inflammatory reaction based published research. This study identified aberrant osteoclast differentiation and other pathways were involved in SAPHO syndrome. This finding may give insight into the understanding of pathogenic genes of SAPHO and provide the basis for SAPHO research and treatment.

## Introduction

SAPHO (synovitis, acne, pustulosis, hyperostosis, and osteitis) syndrome, with the clinical manifestations including auto-inflammatory osteoarticular disorders and dermatological conditions, is a rare disease with an estimated prevalence of less than 1 in 10,000 (Magrey and Khan, 2009). It was first reported by the rheumatologist Chamot in 1987 (Kerrison et al., 2004); however, its etiology is still unknown. Previous research reported that the dysregulation of interleukin-1 (IL-1) signaling promoted sterile osteomyelitis in Pstpip2-deficient mice (Ferguson et al., 2006; Sharma and Ferguson, 2013). However, no specific variants were found using genetic screening in the *PSTPIP1, PSTPIP2, NOD2* or *LPIN2* genes in SAPHO samples (Hurtado-Nedelec et al., 2010; Colina et al., 2012; Guo et al., 2019). There are several factors considered to have the role in the development of SAPHO syndrome, including *Propionibacterium acnes* infection (Kotilainen et al., 1996; Colina et al., 2007), impaired immune responses, over-activated TH17 axis (Firinu et al., 2014). It was reported the proportion and absolute counts of Th17 cells in untreated SAPHO patients were significantly higher than in healthy controls, and the proportion and absolute counts of NK cells were significantly reduced in SAPHO patients compared with controls (Xu et al., 2019). Apart from these, the inflammatory factors including IL-18, IL-6, IL-8, IL-17A, TNF-α, and IL-1β were higher in SAPHO patients compared with healthy controls (Przepiera-Bedzak et al., 2016; Zhang et al., 2019). To date, no evidence-based therapeutic option has been proposed because of the elusive pathogenesis of this disease. Actual major therapeutic drugs are glucocorticoids, bisphosphonates, non-steroidal anti-inflammatory drugs (NSAIDs), disease-modifying antirheumatic drugs (DMARDs) to biologics, and antibiotics (Yang et al., 2018).

Genome-wide association studies (GWASs) has showed remarkable success in detecting the genetic factors of complex diseases by identifying multiple variants associated with complex clinical phenotypes (International Multiple Sclerosis Genetics Consortium, 2013). A commonly mentioned strategy in GWASs involves the evaluation of individual markers by setting a genome-wide significance cutoff *p*-value assuming-independence between markers. In this study, we set up a GWAS study in SAPHO patients, followed by pathway-based analyses of GWAS data that focused on the integrated effects of numerous loci, each making a small direct contribution to estimate of disease susceptibility, which might provide understanding the genetic basis of chronic diseases (International Multiple Sclerosis Genetics Consortium, 2013). The GWAS findings were further validated using whole exome sequencing (WES) to discover genetic variants and abnormal pathways involved in SAPHO patients.

## Materials and Methods

### Patients and Study Design

The GWAS study contained 52 SAPHO patients and 124 healthy controls (detailed characteristics are shown in Table 1). All individuals were enrolled from the Beijing Chaoyang Hospital, and were ethnically and geographically matched. SAPHO syndrome was diagnosed according to the Kahn criteria (Kahn et al., 1994). The study was approved by the Ethics Committee of the National Research Institute for Family Planning, and all participants provided written informed consent for participation in this study. The WES study was composed of 16 SAPHO patients (6 males, 10 females; mean age, 41.4 ± 0.08 years, range 33 to 72 years; 12 patients diagnosed with ACW + S + PS (ACW, anterior chest wall; S, spine; PS, peripheral skeleton), 3 patients diagnosed with ACW + S, 1 patient diagnosed with ACW + PS) and 15 healthy controls (sex and age matched).

**TABLE 1:** General characteristics SAPHO patients in this study.

Characteristics	No. = 52	No. = 124
**Sex**		
Male	18 (34.6%)	47 (37.9%)
female	34 (65.4%)	77 (62.1%)
Age	43.29	46.95
**Skin manifestations**		
None	3 (5.8%)	
PPP	43 (82.7%)	
SA	2 (3.8%)	
PPP + SA	2 (3.8%)	
PPP + PV	2 (3.8%)	
**Osteoarticular symptoms**		
ACW	1 (1.9%)	
ACW + S	18 (34.6%)	
ACW + PS	14 (26.9%)	
ACW + S + PS	19 (36.5%)	

*PPP, palmoplantar pustulosis; SA, severe acne; PV, psoriasis vulgaris; ACW, anterior chest wall; S, spine; PS, peripheral skeleton.*

### DNA Isolation

Blood samples were collected from the peripheral blood of individuals into tubes containing EDTA. DNA extraction was carried out using the RelaxGene Blood DNA System Kit (Tiangen Biotech, Beijing, China) according to the manufacturer’s instructions. For the GWAS, all samples were genotyped individually using Illumina Infinium OmniZhongHua-8v1-3_A1 by the BioMiao Biological Technology Company (Beijing, China). For the WES, 3 μg of purified gDNA was fragmented to 180–280 bp and subjected to DNA library creation using established Illumina paired-end protocols. The Agilent SureSelect Human All ExonV6 Kit (Agilent Technologies, Santa Clara, CA, United States) was used for exome capture according to the manufacturer’s instructions. The Illumina Novaseq 6000 platform (Illumina Inc., San Diego, CA, United States) was utilized for genomic DNA sequencing by Novogene Bioinformatics Technology Co., Ltd (Beijing, China) to generate 150-bp paired-end reads with a minimum coverage of 10× for ∼99% of the genome (mean coverage of 100×).

### Quality Control and Data Mining

**FIGURE 1:**
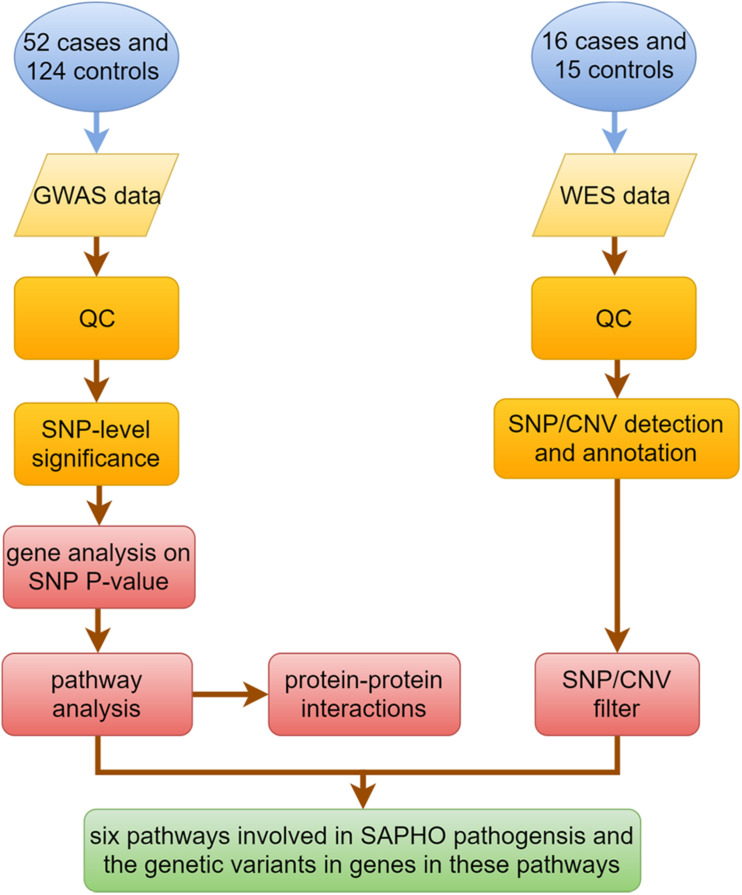
Flow chart of data mining based on GWAS and WES data for SAPHO syndrome.

Figure 1 shows the key steps in our analysis method. For the GWAS study, quality control (QC) and data analysis were performed using the software packages R version 3.6.0^1^ and PLINK version 1.90 beta^2^. Genotype data were cleaned before analysis by excluding SNPs or individuals that did not fulfill the QC criteria, which included: SNP call proportion ≥ 95%, subject completeness proportion ≥ 95%, SNP minor allele frequency ≥ 0.01, and SNP conformity with Hardy-Weinberg equilibrium expectations (*P* ≥ 0.01 in controls). A comparison of cases and controls was made using Pearson’s chi-square tests or Fisher’s exact test. Because this study examined the functional relationships of genes and proteins, we considered gene-level significance rather than that of single SNP in the traditional GWAS studies. To that end, SNPs in the GWAS were mapped to functional genes according to SNP locations and gene locations by MAGMA software (v1.07beta) (de Leeuw et al., 2015). In order to capture gene regulatory regions, gene boundaries were defined as 5 kb beyond the 5′- UTRs and 1.5 kb beyond 3′-UTRs of each gene. Gene analysis on SNP *P*-value data was performed by MAGMA and candidate genes were listed according to the gene *P*-value. *P* < 0.05 was considered statistically significant. Genes with *P* < 0.05 were selected for pathway analysis by DAVID software (v6.8) (Huang da et al.,2009a,b), and protein-protein interactions (PPI) by String software (v11) (Szklarczyk et al., 2019).

For WES, after quality control (QC) and preprocessing of sequencing data, the clean data in fastq format was aligned to the human reference genome hg19 (GRCh37) using the Burrows-Wheeler Aligner (bwa) (Li and Durbin, 2009) along with Samtools (Li et al., 2009). Single nucleotide variants (SNVs) and indels were detected with the best-practices GATK/Picard Pipeline (McKenna et al., 2010; Van der Auwera et al., 2013). The VCF data of all samples were merged by bcftools software for further analysis. Annotation was performed using Ensembl Variant Effect Predictor (v91.3) (McLaren et al., 2016) and ANNOVAR (Wang et al., 2010). The annotation information used for further filtering included minor allele frequencies from public databases, deleteriousness and conservation scores, assessment of the likely pathogenicity of variants and consequence of every single variant identified. After preliminary filtering, we extracted the SNPs in genes involved in disease-related pathways selected by pathway analysis. The copy number variants (CNVs) were detected with ExomeDepth software (Plagnol et al., 2012) after being processed by Samtools and annotated with AnnotSV (Geoffroy et al., 2018).

## Results

### Data Analysis of GWAS

This GWAS study contained 52 SAPHO patients (18 males, 34 females; mean age 43.29 years) and 124 controls (47 males, 77 females; mean age 46.95 years). The number of SNPs were reduced from 878,000 to 802,276 after filtering for low call rate (<90%), minor allele frequency (<0.01) and deviation from Hardy-Weinberg equilibrium (*P* < 0.00001). Six samples were deleted due to quality control, finally 49 cases and 121 controls were left for subsequent analysis. The mean genotyping rate in the remaining individuals was 99.73%.

**FIGURE 2:**
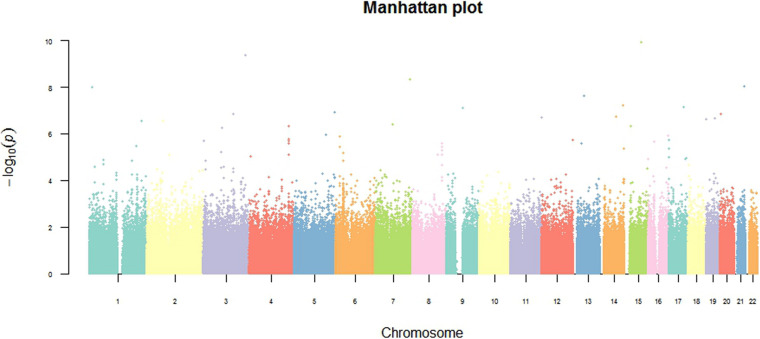
Genome-wide overview of GWAS findings. The Manhattan plot shows genome-wide association analysis of 802,276 single-nucleotide polymorphisms (SNPs) in 49 SAPHO patients and 121 control subjects. The 2 log10 (*P*-value) for each SNP is plotted against their chromosomal position. All statistical tests were two-sided.

To detect associations, we performed a preliminary analysis by Pearson’s chi-square test. Results were adjusted by multiple test correction and then rank ordered on the basis of their *P* values. Overall, 40,588 SNPs in genomic regions were associated with *P* < 0.05 without correction, 84 SNPs were associated with *P* < 5.6 × 10^–7^, and only 9 SNPs met the expected cut-off *P*-value (*P* < 6.24 × 10^–8^, Figure 2). Among 9 SNPs, rs4505038 was located in the intron region of the peroxisomal biogenesis factor 16 gene (*PEX16*), rs2243861 was located in the intron region of the IQ motif containing with AAA domain 1 like gene (*IQCA1L*), and the other 7 SNPs (rs4897770, rs12442139, rs13062589, rs2850133, rs10927436, rs9567768, and rs8007562) were mapped to genomic regions with no known functional genes. Based on previously published literature, none of the 9 SNPs had an association with SAPHO syndrome or other inflammatory disease. Next, we lowered the significance threshold and 84 SNPs with a *P*-value below 5.6 × 10^–7^, a level roughly 10-times the expected threshold, were selected for further analysis. However, no further SNPs or genes were identified (Table 2). Given the complex symptoms and etiology of SAPHO syndrome, we inferred no single genetic variant accounted for this entire complicated syndrome.

**TABLE 2:** Partial SNPs identified in this study (*P* < 5.6 × 10^–7^).

CHR	SNP	Map Info.	Alleles	Gene	Mutation (s)	P value	OR
11	rs4505038	45,934,697	[T/G]	*PEX16*	Intron variant	7.64E–13	7.25
10	rs4897770	133,579,416	[T/C]			1.54E–11	0.08929
15	rs12442139	74,245,486	[T/C]			1.18E–10	0.1608
3	rs13062589	183,195,805	[T/C]			4.05E–10	0.1725
7	rs2243861	150,889,775	[T/C]	*IQCA1L*	Intron variant	4.46E–09	0.06295
21	rs2850133	44,041,365	[T/C]			9.37E–09	0.2018
1	rs10927436	14,763,230	[A/C]			1.01E–08	0.1324
13	rs9567768	47,586,212	[T/C]			2.36E–08	0.2178
14	rs8007562	100,653,848	[A/G]			6.2E–08	4.364
17	rs78395560	64,995,455	[A/G]	*CACNG4*	Intron variant	6.84E–08	4.113
9	rs28461568	71,626,508	[A/T]			7.6E–08	0.09177
5	rs3812081	177,549,334	[A/G]	*N4BP3*	3 Prime UTR variant	1.24E–07	0.112
20	rs3812081	3,776,368	[T/C]	*CDC25B*	3 Prime UTR variant	1.42E–07	0.05509
3	rs118184987	131,095,476	[A/G]	*LOC339874*	Non-coding transcript variant	1.46E–07	10.2
14	rs2280792	73,711,394	[A/G]	*PAPLN*	Missense_S33G	1.85E–07	0.2284
11	rs4937861	133,864,279	[A/G]			1.94E–07	0.09827
19	rs10401843	39,449,513	[A/G]	*FBXO17*	Genic upstream transcript variant	2.09E–07	0.2246
19	rs7508251	401,714	[A/G]			2.41E–07	0.2321
2	rs1809265	72,013,617	[T/C]			2.73E–07	0.2115
1	kgp11029205	227,462,940	[A/T]	*CDC42BPA*	Intron variant	2.82E–07	0.08025
7	rs574637	73,752,699	[T/C]	*CLIP2*	Intron variant	3.79E–07	0.119
4	rs6847701	170,781,319	[A/G]			4.72E–07	0.2703
15	rs2279486	29,400,125	[T/C]	*APBA2*	Intron variant	4.76E–07	3.604
3	rs1461616	81,420,196	[A/C]			5.55E–07	0.1513

As in other GWAS studies of this kind, many SNPs that did not reach the statistical significance level were abandon in further analysis. There is a certain proportion of rejected associations that are actually false negative; meanwhile, many studies have showed some significant combinations of gene markers with only limited association if they were involved in the same biological pathway or molecular mechanism. Compared to single-locus associations identified by classical genome-wide analysis, this type of analysis is useful for identifying pathways and networks involved in disease susceptibility in accordance with current models of pathogenesis, as well as identifying statistically over-represented but unexpected pathways responsible for novel disease mechanisms (Baranzini et al., 2009; Ritchie, 2009; International Multiple Sclerosis Genetics Consortium, 2013).

### Pathway Analysis of GWAS Data

**FIGURE 3:**
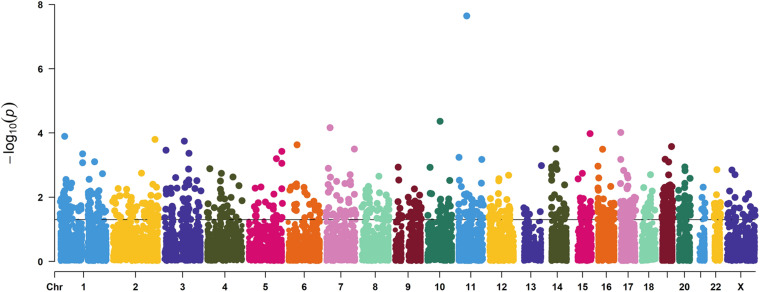
A Manhattan plot showing the gene level *P* values of GWAS used in this study. Genes in each chromosome are represented by different colors.

**FIGURE 4:**
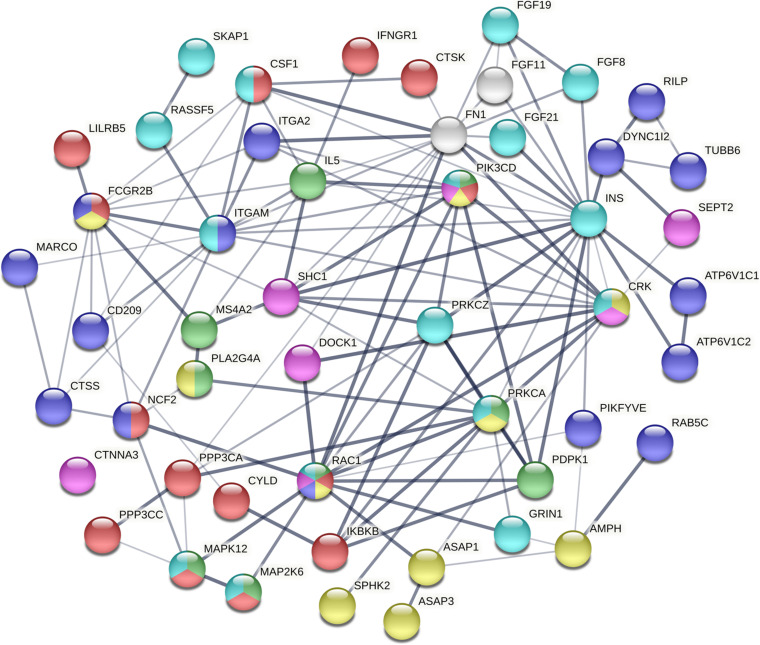
The interaction of mutated genes in SAPHO associated pathways identified in this study. The mutated genes of different pathways are shown by different colors. Each color represents a unique pathway and line thickness indicates the strength of data support (red, osteoclast differentiation; purple, phagosome; green, Fc epsilon RI signaling pathway; yellow, Fc gamma R-mediated phagocytosis; pink, Bacterial invasion of epithelial cells; blue, Rap1 signaling pathway).

To dissect the pathways involved in SAPHO disease, we proposed a pathway-oriented analysis of the GWAS result. We analyzed a list of differentially expressed genes and a *P*-value for each gene was performed on SNP that indicated the strength of the gene-disease associations. Many SNPs that were not annotated within gene regulatory regions were excluded from the present analysis. In this step, we computed gene-wise *P*-values for 18,151 genes for the GWAS, of which, 891 genes reached the significance threshold of *P* < 0.05 (Figure 3). For the pathway enrichment analysis, we mapped these screened genes to 15 KEGG pathways (Table 3) including osteoclast differentiation, glycosphingolipid biosynthesis, amyotrophic lateral sclerosis, cell-matrix interactions, and inflammatory associations, with a default threshold of the EASE Score (a modified Fisher Exact *P*-Value). Because of the multiple roles of some genes and complex interactions in cellular activities between protein pathway networks, candidate genes were always involved in different pathways. For example, mitogen-activated protein kinase 12 (*MAPK12*) gene, an important transduction factor of extracellular signals, was involved in osteoclast differentiation, amyotrophic lateral sclerosis (ALS), Fc epsilon RI signaling pathway, VEGF signaling pathway, Rap1 signaling pathway, MAPK signaling pathway, and T cell receptor signaling pathway (Figure 4). Previous studies report contradictory information regarding which pathways might be related to inflammatory reactions or symptoms of SAPHO syndrome, therefore, it is important to preclude potential misleading pathways. Based on published studies of identified pathways, we inferred osteoclast differentiation pathway (*P* = 0.002954, 15 genes involved), phagosome (*P* = 0.009788, 15 genes involved), Fc epsilon RI signaling pathway (*P* = 0.013035, 9 gene involved), Rap1 signaling pathway (*P* = 0.035055, 17 genes involved), Fc gamma R-mediated phagocytosis pathway (*P* = 0.040769, 9 genes involved), and bacterial invasion of epithelial cells pathway (*P* = 0.069759, 8 genes involved) were highly correlated with SAPHO syndrome based on bone and skin manifestations of SAPHO. Other pathways may affect the pathological process of SAPHO syndrome as well, but their direct association requires further research.

**TABLE 3:** KEGG pathways Identified in SAPHO samples (*P* < 0.10).

	Pathway	*P* Value	Gene No.	Genes
1	Osteoclast differentiation	0.002954	15	*NCF2, CSF1, PIK3CD, CYLD, CTSK, FCGR2B, MAPK12, LILRB5, LILRA3, RAC1, PPP3CC, PPP3CA, IKBKB, IFNGR1, MAP2K6*
2	Glycosphingolipid biosynthesis-lacto and neolacto series	0.006194	6	*B3GALT2, B3GALT1, ST3GAL4, FUT6, ST8SIA1, FUT1*
3	Amyotrophic lateral sclerosis (ALS)	0.007868	8	*DERL1, MAPK12, GRIN1, RAC1, PPP3CC, PPP3CA, NEFL, MAP2K6*
4	Phagosome	0.009788	15	*RILP, NCF2, RAB5C, ITGA2, CTSS, ITGAM, MARCO, ATP6V1C1, ATP6V1C2, FCGR2B, CD209, RAC1, PIKFYVE, TUBB6, DYNC1I2*
5	Amphetamine addiction	0.01097	9	*PRKCA, PRKACG, GRIN1, MAOB, PPP3CC, CREB3L1, PPP3CA, CACNA1C, SIRT1*
6	Fc epsilon RI signaling pathway	0.013035	9	*PRKCA, PLA2G4A, PDPK1, IL5, MAPK12, PIK3CD, RAC1, MS4A2, MAP2K6*
7	VEGF signaling pathway	0.022293	8	*PRKCA, PLA2G4A, MAPK12, SPHK2, PIK3CD, RAC1, PPP3CC, PPP3CA*
8	Rap1 signaling pathway	0.035055	17	*PRKCA, FGF19, PRKCZ, FGF8, CSF1, GRIN1, PIK3CD, FGF11, FGF21, SKAP1, ITGAM, RASSF5, MAPK12, INS, RAC1, CRK, MAP2K6*
9	Fc gamma R-mediated phagocytosis	0.040769	9	*PRKCA, FCGR2B, SPHK2, PIK3CD, RAC1, ASAP1, ASAP3, CRK, AMPH*
10	Insulin signaling pathway	0.056048	12	*PRKACG, PRKCZ, PDPK1, INS, HKDC1, PIK3CD, PRKAG2, PRKAR1A, SHC1, IKBKB, CRK, PCK1*
11	Bacterial invasion of epithelial cells	0.069759	8	*DOCK1, SEPT2, PIK3CD, RAC1, SHC1, CRK, CTNNA3, FN1*
12	Type II diabetes mellitus	0.070714	6	*PRKCZ, INS, HKDC1, PIK3CD, IKBKB, CACNA1C*
13	MAPK signaling pathway	0.080198	18	*PRKCA, FGF19, FGF8, FGF11, CACNG4, FGF21, CDC25B, PRKACG, RPS6KA5, PLA2G4A, MAPK12, RAC1, PPP3CC, PPP3CA, IKBKB, CACNA1C, CRK, MAP2K6*
14	Taurine and hypotaurine metabolism	0.089925	3	*BAAT, GGT7, GADL1*
15	T cell receptor signaling pathway	0.093064	9	*PDPK1, IL5, MAPK12, PIK3CD, CTLA4, PPP3CC, PPP3CA, IKBKB, CD28*

### Detect Genetic Variants Based on WES Data

Genome-wide association study has limited power in identifying low frequency and rare causal genetic variants with greater penetrance involved in complex diseases. To verify the association between aberrant signaling pathways and SAPHO syndrome, we performed WES analysis for 16 patients diagnosed with SAPHO and 15 healthy individuals. Results showed the coverage of target region was 99.91%, mean depth on target region was 181.52 ± 21.33× and the target coverage with at least 20× was 94 ± 2% (Table 4). After filtering, sequencing resulted in 176,181 SNP/INDEL and 55.31 CNV variants in each SAPHO cases and 179,145.5 SNP/INDEL and 33.57 CNV variants in healthy control samples (Table 5). First of all, we tried to identify the shared genetic variants or genes in SAPHO and control groups. Again, the result of the WES was inconsistent between individual samples, no single genetic variant or gene was highly conserved in more than three cases when compared with healthy controls. Next, we aligned these variants to KEGG pathways identified in the GWAS analysis. The result showed each SAPHO sample had at least one aberrant pathway involved: 15 samples had gene aberrations in osteoclast differentiation pathway (genetic variants were found in *CSF1*, *FCGR2B*, *PIK3CD*, *MAP2K6*, *LILRB5*, *PPP3CA* and *MAPK12*, CNV aberrations were found in *LILRA3*, *FCGR2B*, and *CTSK* genes); 7 samples had gene aberrations in the phagosome pathway (genetic variants were found in *ITGA2*, *MARCO*, *FCGR2B* and *CD209*, CNV aberrations were found in the *CTSS* gene); 6 samples had gene aberrations in the Fc epsilon RI signaling pathway (genetic variants were found in *PIK3CD*, *MAP2K6* and *MAPK12*, CNV aberrations were found in the *PDPK1* gene); 7 samples had gene aberrations in Rap1 signaling pathway (genetic variants were found in *MAP2K6*, *MAPK12*, *CRK*, *CSF1*, and *GRIN1*); 5 samples had gene aberrations in the Fc gamma R-mediated phagocytosis pathway (genetic variants were found in *FCGR2B*, *PIK3CD*, *AMPH*, *SPHK2*, and *CRK*); 11 samples had gene aberrations in the Bacterial invasion of epithelial cells pathway (genetic variants were found in *PIK3CD*, *DOCK1*, *CRK*, *CTNNA3*, and *FN1*) (Tables 6, 7).

**TABLE 4:** Basic results of the WES used in this study.

Mapped	Raw reads	Raw data (G)	Raw depth (x)	Effective	Q30	Sequencing depth on target	Fraction of target covered with at least 20x
99.91 ± 0.01	36577912 ± 4299644	10.97 ± 1.29	181.52 ± 21.33	98.87 ± 0.36	93.35 ± 0.57	109.64 ± 7.16	94 ± 2%

**TABLE 5:** SNP/INDEL and CNV identified by WES in this study.

	SAPHO		CONTROL	
	Mean	Variance	Mean	Variance
**SNP/INDEL**				
Exonic	21891.57	204.84	21895.23	274.97
Intronic	77119.71	4752.78	78990.39	5347.49
UTR3	4009.43	169.02	4038.92	193.51
UTR5	2556.57	133.08	2539.85	99.65
Intergenic	32708.71	4708.29	33669.54	4700.08
NcRNA_exonic	2895.71	111.19	2914.69	66.05
NcRNA_intronic	6965.71	582.41	7094.92	646.83
Upstream	2311.43	335.13	2271.92	202.45
Downstream	1305.29	149.63	1324.08	150.29
Splicing	2425.14	41.93	2413.15	59.72
NcRNA_splicing	100.14	7.43	97.54	4.27
Synonymous SNV	11190.14	118.74	11201.46	149.19
Missense SNV	10153	108.28	10157.31	140.97
Stopgain	73.14	6.82	74.46	6.46
Stoploss	8.29	0.49	8.31	1.38
Unknown	467	20.12	453.69	33.86
**CNV**				
dup	20.77	28.02	24.14	28.73
del	34.54	88.19	9.43	8.21

**TABLE 6:** The clinical features of 16 SAPHO patients and genetic variants corresponding to GWAS results (*P* < 6.24 × 10^–8^).

Sample	Gender	Age	Symptoms		Gene	Location	Mutation	
			Skin	Osteoarticular			Codons	Amino_acids
S1	M	41	PPP	ACW + S + PS				
S2	M	46	PPP	ACW + S + PS	*CLIP2*	Splice_region_variant	NM_003388.4:c.2421 + 7del	
S3	M	33	PPP	ACW + S + PS				
S4	M	37	PPP	ACW + S + PS				
S5	F	72	PPP	ACW + S + PS				
S6	F	50	PPP	ACW + S + PS				
S7	F	52	PPP	ACW + S + PS				
S8	F	61	PPP	ACW + S + PS	*PAPLN*	Missense_variant	NM_173462.3:c.653C > T	p.A218V
S9	F	33	PPP	ACW + S + PS	*CLIP2*	Missense_variant	NM_003388.4:c.2627G > A	p.R876H
S10	M	35	PPP	ACW + S + PS				
S11	F	33	PPP	ACW + S + PS				
S12	M	34	PPP	ACW + S				
S13	F	52	PPP	ACW + S + PS				
S14	F	49	PPP	ACW + S	*CACNG4*	3_prime_UTR_variant	NM_014405.3:c.*875G > A	
					*PAPLN*	Missense_variant	NM_173462.3:c.985A > C	p.N329H
					*APBA2*	Missense_variant	NM_005503.3:c.34G > A	p.G12S
S15	F	41	PPP	ACW + S	*FBXO17*	Missense_variant	NM_148169.2:c.760T > C	p.Y254H
S16	F	33	PPP	ACW + PS				

PPP, palmoplantar pustulosis; SA, severe acne; PV, psoriasis vulgaris; ACW, anterior chest wall; S, spine; PS, peripheral skeleton. “*” represents for “Untranslated Region, (UTR)”.

**TABLE 7:** Genetic variants identified in 6 pathways of 16 SAPHO patients.

Sample	Osteoclast differentiation pathway	Phagosome pathway	Fc epsilon RI signaling pathway	Rap1 signaling pathway	Fc gamma R-mediated phagocytosis	Bacterial invasion of epithelial cells pathway
S1	*FCGR2B* c.169C>T, p.Q57Ter *PIK3CD* c.1953C>T, p.L651%3D (splice_region_variant) *LILRA3* Copy No.= 4	*ITGA2* c.*95dup (3′ UTR); *MARCO* c.1143dup, p.G382RfsTer35; *FCGR2B* c.169C>T,p.Q57Ter; *CD209* c.1211C>T, p.A404V	*PIK3CD*:c.1953C>T, p.L651%3D (splice_region_variant);		*FCGR2B* c.169C>T, p.Q57Ter; *PIK3CD* c.1953C>T, p.L651%3D (splice_region_variant); *AMPH* c.1157C>T, p.T386M	*PIK3CD* c.1953C>T,p.L651%3D (splice_region_variant)
S2	*MAP2K6* c.*149C>A, (3′-UTR) *LILRA3* Copy No.= 4 *FCGR2B* Copy No.= 3	*ITGA2* c.*95dup (3′-UTR)	*MAP2K6* c.*149C>A (3′-UTR)	*MAP2K6* c.*149C>A, (3′ UTR)		*DOCK1* c.1201+4C>T (splice_region_variant); c.1912+8C>T (splice_region_variant)
S3	*LILRB5* c.196C>T, p.P66S *LILRA3* Copy No.= 0					
S4	*PPP3CA* c.1514C>G, p.S505Ter *LILRA3* Copy No.= 0	*ITGA2* c.*95dup (3′-UTR)				
S5	*LILRA3* Copy No.= 0	*ITGA2* c.*95dup (3′-UTR)			*SPHK2* c.1678C>T, p.R560C	*DOCK1* c.1912+8C>T (splice_region_variant)
S6	*CSF1* c.523T>C, p.F175L *LILRA3* Copy No.= 0			*CSF1* c.523T>C, p.F175L		
S7	*LILRA3* Copy No.= 0		*PDPK1* Copy No.= 1			*DOCK1* c.1201+4C>T (splice_region_variant); c.1912+8C>T (splice_region_variant)
S8	*LILRA3* Copy No.= 0	*MARCO* c.419A>G, p.N140S *CD209* c.-35C>A	*MAPK12* c.*104C>T (3′-UTR) *PDPK1* Copy No.= 0	*CRK* c.*549C>T (5′-UTR) *GRIN1*: c.*8C>T (3′ UTR)	*CRK* c.*549C>T (5′ UTR)	*CRK* c.*549C>T (5′ UTR)
S9	*MAPK12* c.*104C>T (3′-UTR); *MAPK12* c.887C>T, p.A296V *LILRA3* Copy No.= 0	*ITGA2* c.*95dup (3′-UTR)	*MAPK12* c.*104C>T (3′-UTR); *MAPK12* c.887C>T, p.A296V	*MAPK12* c.*104C>T (3′-UTR); *MAPK12* c.887C>T, p.A296V		*DOCK1* c.1171G>A, p.A391T
S10	*LILRA3* Copy No.= 0 *CTSK* Copy No.= 3	*CTSS* Copy No.= 3				*CTNNA3* c.1450C>T, p.R484C
S11	*CSF1* c.1223T>C, p.L408P; c.1466T>C, p.F489S *PIK3CD* c.1366A>G, p.T456A			*CSF1* c.1223T>C, p.L408P; c.1466T>C, p.F489S	*PIK3CD* c.1366A>G, p.T456A; *AMPH* c.1487A>C, p.K496T	*DOCK1* c.5569G>A, p.A1857T; *PIK3CD* c.1366A>G, p.T456A; *CTNNA3* c.1787G>A, p.S596N *FN1* c.5878G>A, p.V1960I; c.2449A>C, p.T817P; c.44A>T, p.Q15L
S12	*CSF1* c.523T>C, p.F175L; c.1223T>C, p.L408P; c.1312G>A, p.G438R; c.1118T>C, p.F373S			*CSF1* c.523T>C, p.F175L; c.1223T>C, p.L408P; c.1312G>A, p.G438R; c.1118T>C, p.F373S		*DOCK1* c.5569G>A, p.A1857T; c.5377G>A, p.A1793T *FN1* c.5878G>A, p.V1960I; c.2449A>C, p.T817P; c.44A>T, p.Q15L
S13	*CSF1* c.1223T>C, p.L408P; c.1118T>C, p.F373S			*CSF1* c.1223T>C, p.L408P; c.1118T>C, p.F373S		*DOCK1* c.5569G>A, p.A1857T; *FN1* c.5878G>A, p.V1960I; c.2449A>C, p.T817P; c.44A>T, p.Q15L
S14					*AMPH* c.-143C>G (5′-UTR)	
S15	*LILRA3* Copy No.= 0		*PDPK1* Copy No.= 1			
S16	*LILRA3* Copy No.= 4 *FCGR2B* Copy No.= 3					*DOCK1* c.1201+4C>T (splice_region_variant); c.1912+8C>T (splice_region_variant); *CTNNA3* c.398C>T, p.T133M

“*” represents for “Untranslated Region, (UTR)”.

## Discussion

SAPHO syndrome is a systemic and recurrent disease with unknown etiology, characterized by chronic inflammatory osteoarticular lesions and dermatological disorders. The diagnosis and treatment of this rare disease have been limited by its complex etiology and phenotypic heterogeneity. In the last decade, many studies have demonstrated that comprehensive analyses of GWAS data and protein-protein interaction (PPI) networks can provide valuable biological clues (Cong et al., 2017). In this study, to identify genetic variants of SAPHO syndrome, we performed a novel genome-wide network-based integrative analysis of SAPHO syndrome based on GWAS and WES data. Using a basic GWAS analysis after filtering process, we found 9 SNPs (rs4505038, rs4897770, rs12442139, rs13062589, rs2850133, rs10927436, rs9567768, rs2243861, and rs8007562) that met the expected significance threshold (*P* < 6.24 × 10^–8^). Among them, 7 SNPs located in the regions without known functional genes, rs4505038 (*PEX16*) and rs2243861 (*IQCA1L*) located in the introns of known genes. *Pex16* plays an critical role in adipose tissue peroxisomal biogenesis, and mice deficient for the *Pex16* gene showed increased diet-induced obesity and impaired thermogenesis ability without skin or osteoarticular manifestations (Suzuki et al., 2001; Park et al., 2019). The *IQCA1L* gene is specifically expressed in the testis and has not been reported with immunity (Gaudet et al., 2011). Based on previous studies, none of the 9 SNPs had an association with SAPHO syndrome or other inflammatory diseases. Then we lowered the significance threshold to approximately 10-times the expected threshold, 84 SNPs with a *P*-value below 5.6 × 10^–7^ were selected for further analysis. However, no valuable SNP or candidate genes were identified.

Given the complex symptom and etiology of SAPHO syndrome, we speculate that no single genetic variant accounts for all the complicated manifestations of this disease. Thus, in the following analysis, we reanalyzed the GWAS data by adopting pathway and network-based analysis. We found several pathways were altered in SAPHO samples, and six of these had evidence with skin, osteoarticular manifestations of SAPHO syndrome or inflammatory reactions, including osteoclast differentiation pathway, phagosome pathway, Fc epsilon RI signaling pathway, Rap1 signaling pathway, Fc gamma R-mediated phagocytosis pathway, and bacterial invasion of epithelial cells pathway.

The osteoclast differentiation pathway, a key regulator of resorption and formation of bone tissue, was the most significant aberrant pathway in SAPHO patients. Previous studies reported disruption of the osteoclast differentiation or function leads to inhibited bone resorption, which further can result in bone marrow deficiency and no teething (Grigoriadis et al., 1994; Kong et al., 1999). On the contrary,enhancement of osteoclast differentiation or function in patients with osteoporosis and metastatic bone cancer resulted in the decrease of bone mass and destruction of bone, respectively (Miyamoto, 2011). Some important signaling molecules are essential for the correct fulfillment of osteoclastogenesis, for example, monocyte colony-stimulating factor (M-CSF) exert a proliferative and survival effect on early pre-monocyte phase and the entire process, respectively (Boyle et al., 2003; Edwards and Mundy, 2011; Anesi et al., 2019). The function of the second signaling molecule receptor activator NF-κB ligand (RANKL) is differentiation in the late post-monocyte phase of the process that is necessary to transform monocytes into osteoclasts (Takayanagi,2007b,a,c; Kim and Kim, 2016; Kim et al.,2016a,b; Anesi et al., 2019). Osteoarticular involvement, a characteristic sign of disease, was observed in nearly all SAPHO patients and mainly involved the anterior chest wall and lumbosacral and peripheral skeletal regions (Cao et al., 2019). Zhang et al. (2019) reported RANKL levels were significantly higher in active SAPHO patients than in non-active or healthy samples, suggesting the aberrant osteoclast differentiation pathway plays pivotal role in the pathology of SAPHO. Our findings reconfirmed the foregoing conclusion. In addition to the molecules mentioned above, mary other signaling molecules play important role in regulating osteoclastic differentiation process as well. Osteoclastogenic cytokines are represented by inflammatory cytokines including tumor necrosis factor α (TNF-α), interleukin-1 (IL-1), IL-6, IL-7, IL-8, IL-11, IL-15, IL-17, IL-23, and IL-34. Anti-osteoclastogenic cytokines are represented by IFN-α, IFN-β, IFN-γ as well as IL-3, IL-4, IL-10, IL-12, IL-27, and IL-33 (Amarasekara et al., 2018). Published research reported some inflammatory factors including IL-1β, IL-17A, IL-6, IL-8, IL-18, and TNF-α were higher in SAPHO patients than in healthy controls (Przepiera-Bedzak et al., 2016; Zhang et al., 2019), based on these findings, it is plausible that the combined actions of elevated cytokines and a disrupted osteoclast differentiation pathway might aggravate bone devastation and reconstruction, resulting in the osteoarticular symptoms.

Phagocytosis is an evolutionarily ancient process whereby cells engulf large particles. It is an important core mechanism in some immune processes, including defense against infectious agents, inflammation, tissue remodeling, and antigen degradation and presentation (Dean et al., 2019). Phagocytic cells such as monocytes and macrophages participate in host defense by forming phagosomes. During phagocytosis, the membrane on the surface of a phagocyte forms a phagosome when the receptors on it bind to the ligands on the surface of the particle surface. After its formation, the new phagosome gradually acquire digestive properties. In the process of phagosome maturation, there are other membrane organelles involved, including circulating endosomes, late endosomes, and lysosomes. By fusing lysosomes, phagosomes can activate enzymes and lower the pH value in the lumen that eventually degrades phagocytized micro-organisms into fragments (Kanehisa et al., 2017). Accordingly, disruptions to this process cause some bacteria such as *Mycobacterium tuberculosis* to escape bacterial killing and survive within host phagocytes (Ehrt and Schnappinger, 2009; Kanehisa et al., 2017). In this study, two phagocytosis-related pathways (phagosome, *P* = 0.0098, 15 genes; Fc gamma R-mediated phagocytosis, *P* = 0.041, 9 genes) were highly associated with SAPHO syndrome, suggesting phagocytosis has an important role in SAPHO syndrome. James et al. (2010) found the phagocytosis of disease-relevant particles (PMMA, titanium, and silica) inhibited the RANKL-mediated osteoclastogenesis of human monocytes. They demonstrated phagocytosis mediates this effect by down-regulation of RANK and c-Fms, receptors for the essential osteoclastogenic cytokines RANKL and M-CSF (James et al., 2010). However, the mechanisms involved in phagocytosis and SAPHO required further research.

Fc epsilon RI is the specific receptor for IgE, which has an important role in IgE-associated allergic reactions. A cascade of signaling events can be induced by the cross-linking of Fc epsilon RI on mast cells, leading to degranulation, proinflammatory cytokine production, and leukotriene release, which contribute to the emergence of allergic symptomology (Klemm and Ruland, 2006; Kambayashi and Koretzky, 2007). IFN-γ activates mast cells through FceRI to induce PGD2 and LTC4 release, and the subsequent up-regulation of mRNAs for IL-1a, IL-3, IL-8, G-CSF, LIF, CSF1, oncostatin M (OSM), SCF, TGF-β1, IP-10, I-309, MIP-1α, and MIP-1β (Okayama et al., 2001). Our results showed that the Fc epsilon RI signaling pathway was involved in the pathogenesis of SAPHO syndrome (*P* = 0.013035, 9 genes). In accordance, Li et al. reported a SAPHO patient with elevated serum immunoglobulin E levels, and demonstrated methylprednisolone treatment achieve long-term remarkable remission on clinical manifestations (Wang et al., 2019), which is consistent with our finding.

In this study, we found two pathways associated with aberrant cell barrier function in SAPHO patients, Rap1 signaling pathway (*P* = 0.035055, 17 genes) and bacterial invasion of epithelial cells (*P* = 0.069759, 8 genes), suggesting damage to the cell barrier contributes to the complicated manifestation of SAPHO syndrome. The function of the small G-protein Rap1 is to regulate endothelial barrier function controlled by cell–cell adhesion and the actin cytoskeleton. When this process is activated, numerous signaling cascades are induced by Rap1 to enhance the endothelial barrier function. Of note, Rap1 activation results inhibit of Rho to decrease radial stress fibers and activate Cdc42 to increase junctional actin (Pannekoek et al., 2014). These are some studies has proven the above results in human umbilical endothelial cells (Cullere et al., 2005; Citalan-Madrid et al., 2013) and retinal vascular endothelial cells (Ramos et al., 2018). Moreover, Rap1 deletion in mature osteoclasts caused osteopetrosis by reducing talin/β integrin recognition (Zou et al., 2013).

Unlike other immunologically relevant diseases, except chronic multifocal osteomyelitis, SAPHO patients suffer from recurrent demographic manifestations, including palmoplantar pustulosis, psoriasis vulgaris, and severe acne. On the base of the findings in this study, we inferred a single pathway was not responsible for this complicated syndrome, two or more pathways probably act simultaneously. For example, an impaired cell barrier or inflammatory cytokine release induced by allergic reactions might promote the demographic manifestations and elevated inflammatory factors, moreover aberrant phagocytosis and osteoclast differentiation pathways might cause alterations to bone resorption and formation, ultimately leading to osteoarticular deformation. These pathways are closely linked and might affect each other, for example, an impaired cell barrier and pathogen infection or allergic reaction might lead to the over-expression of inflammatory factors, which increase the permeability of the skin or endothelial cells, thus increasing infection. Moreover, the disruption of phagocytosis might allow bacteria to escape and enhance infection.

## Conclusion

In conclusion, this GWAS study combined with pathway-based analysis and WES identified aberrant pathways including the osteoclast differentiation pathway involved in SAPHO syndrome. This finding may provide insights into the pathogenic genes of SAPHO and provide the basis for SAPHO research and treatment. Further studies should be conducted to validate this conclusion in a larger sample size and in other ethnic backgrounds.

## Data Availability Statement

The raw sequence data reported in this article have been deposited in the Genome Sequence Archive (Wang et al., 2017) in BIG Data Center (National Genomics Data Center Members and Partners, 2020), Beijing Institute of Genomics, Chinese Academy of Sciences, under accession number(s) HRA000288. All data is available from the corresponding author upon request.

## Ethics Statement

The studies involving human participants were reviewed and approved by the Ethics Committee of the National Research Institute for Family Planning. The patients/participants provided their written informed consent to participate in this study.

## Author Contributions

RC and YD: planning of the project, analysis and interpretation of data, and drafting the manuscript. MF, YF, and JG: analysis and interpretation of data. YZ, ZC, and FH: collecting of the data. CG and XM: putting forward research ideas and planning of the project. All authors contributed to the article and approved the submitted version.

## Conflict of Interest

The authors declare that the research was conducted in the absence of any commercial or financial relationships that could be construed as a potential conflict of interest.

## Footnotes

1 http://www.R-project.org

2 http://www.cog-genomics.org/plink2/
